# Modeling CO_2_ Adsorption in a Thin Discrete Packing

**DOI:** 10.1021/acs.iecr.3c04231

**Published:** 2024-04-10

**Authors:** Michael Wray, Farida Amrouche, Farid Aiouache

**Affiliations:** School of Engineering, Lancaster University, Lancaster LA1 4YR, U.K.

## Abstract

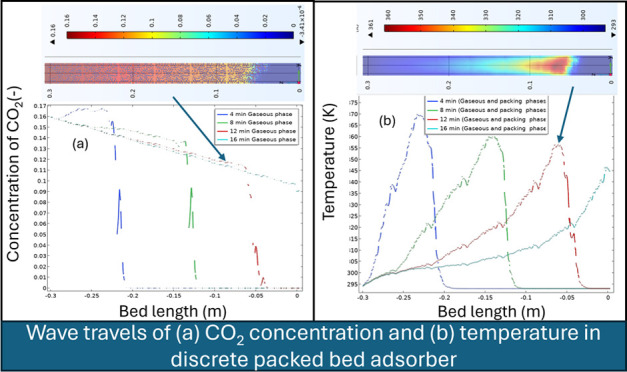

Local dynamics of CO_2_ adsorption in a discrete packing contained in a thin tube was assessed by 3D modeling. Thin tube packed bed adsorbers are currently used over tube structures in thermochemical energy storage systems and atmospheric revitalization of confined spaces. Driven by the interplay between key factors such as the exothermicity and the fluid flow, the advective transport was found less effective than the diffusive one on the breakthrough trends of CO_2_ which displayed significant concentration gradients at both inter- and intraparticle scales. The lack of angular symmetry inside the particles by the reduction in resistance to mass transfer in the area of solid particles exposed to high velocities led to greater convective transports from the bulk of the gaseous phase to the pores. The result of the modeling agreed with the experimental data obtained at the exit of the adsorber, helping reduction in reliance on the empirical dispersion models used in the one-dimensional modeling.

## 1 Introduction

With the current environmental concerns and restrictions placed on emissions, the reduction of anthropogenic pollutants stands as an important potential solution. The Paris agreement has set an ambitious target to mitigate effects of global warming by a reduction of 45% by 2030 in greenhouse (GHG) emissions to reach net zero by 2050.^1^ CO_2_ gas, however, as the largest contributor to the production of these, is still exhibiting an alarming increase of its global concentration. An effective prevention or reduction of its release to the atmosphere will be a strong step in the direction of mitigating GHG effects. An area of focus within the CO_2_ reduction strategies is capture through adsorption in packed bed adsorbers (PBAs). These beds make use of stationary solid particles held within a tubular vessel that captures CO_2_ by means of a physical adsorption, via an ion–dipole interaction with the CO_2_ in a linear orientation, from fluid streams passing through. The process is one of mass-transfer principles and relies on a good surface of capture and high selectivity to CO_2_ of typical gas mixtures. Fundamental understanding of the local adsorption is crucial to design and effectively operate the PBA devices.

An approach to engineering solutions involves a number of approximations [e.g., an averaging into single-dimensional (1D) and two-dimensional (2D) models] which are often specifically tailored to individual operational cases. This approach to engineering design, which is adopted for the generation of key adsorption design parameters, such as the breakthrough curves and the adsorption times, could be complemented by description of the local phenomena taking place at the intra- and interparticle levels of PBAs. The case of the adsorption of CO_2_ is worth investigation as it uses microporous materials of large surface area, and thus the relevance of mass transfer in the pores cannot be ignored. Due to interactions of mass transfer and fluid flow, the adsorption and/or desorption cycles are typically designed by use of approximation models such as the linear driving force (LDF) models associated with breakthrough curves at the exit of the PBA.^2−4^ The LDF models include spatially averaged information on surface adsorption, mass, and heat transfers inside and outside the solid adsorbent. While this term is used variably by the authors, LDF applies to the surface of adsorption by assuming an average concentration within the particles and a uniform temperature. This lumping approach of phase characteristics is associated with the models describing the local rates of adsorption and isotherms at the particle level that are often lost in the final solutions. Such an approach might become insufficient for tube bundle-type adsorbers of low aspect ratio (AR) of tube to particle diameter of PBAs with wall effects on porosity of the bed, leading to nonuniformity of flow, mass, and heat. The tube bundle-type adsorbers are seeing interest in applications to the thermochemical energy storage, atmospheric revitalization, and fine chemistry,^5,6^ anticipating additional knowledge on management of nonuniform mixing of flow. Typically used for applications of adsorption processes to manage impact of the exothermicity and mitigate nonisothermal operations, low AR packed tube bundles or single packed beds are adapted to applications such as the direct air capture, air drying, energy storage, laboratory-scale gas separation, and chromatographic analysis where the operated flow rates are generally of moderate values.^7−9^

One of the keys when carrying out three-dimensional (3D) modeling is the reduced use of lumped models of averaged mass- and heat-transfer coefficients between the adsorbents and fluid in the PBA. Siriwardane et al.^7^ investigated the adsorption of water vapor in a discrete structure using the lattice Boltzmann method. Interesting nonsymmetric phenomena of concentration trends in the PBA were observed at the pore scale, which were caused by an uneven distribution between particles of the macropore voids.^10^ Symmetrical trends around the central axis were, however, observed, despite uneven void concentrations, when slow uptakes in the solid phase occurred. Further observations were extended to nonuniform radial gradients of temperature in regions of low to stagnant flow, affecting breakthrough profiles and times.

The literature is richer, however, with models of adsorption based on the LDF models or deactivation models to account for the contribution of mass transfer to the kinetics of adsorption.^11,12^ These models relied on assumption of homogeneous temperature in the beads while model parameters responsible for flow dispersion such as the transfer coefficients were estimated by a fitting approach of data from laboratory experiments. For instance, Dantas et al.^13^ investigated by process modeling the dual adsorption of CO_2_ and N_2_ inside a PBA of zeolite 13X. The model was based upon the LDF approach and included the thermal effects and allowed access to rate of adsorption inside the particle adsorber by assuming the geometrical profiles of concentration and temperature occurring in large size PBAs. In case of low AR, however, nonaxially symmetric profiles of concentration and temperature are anticipated due to velocity heterogeneity which justifies use of complementary information on the PBA for 1D models.^11^ Knox et al.^2^ recommended use of local data, instead of exit data, due to front-sharpening patterns of concentration driven by the axial dispersion, typically considered constant along the PBA 1D models.

The work undertaken herein is focused on local behavior of CO_2_ adsorption inside a PBA of zeolite using the configuration of a discrete packing. Nanoporous zeolites were selected as they exhibit a physical type of adsorption of CO_2_ and N_2_ with reduced relevance of the kinetics regime of surface adsorption with reference to the associated transport phenomena of heat and mass inside and around the porous surfaces.^14^ Zeolites are one of the favored structures for the postcombustion application due to the structured crystalline framework and the controlled pore size variations.^15^ The crystalline framework offers both chemical and size limitations on adsorbing species, and this means selectivity and capacity, particularly at high temperature or in the presence of contaminants such as water vapors, can be tuned at the user’s preference.^16^

An issue with zeolites in the application of CO_2_ adsorption is the low selectivity between other components in gaseous streams (N_2_, H_2_O, etc.). Besides this, and similar to other physical adsorption-type materials, the capacities are sensibly reduced at an increased temperature, which may pose problems in flue gas treatment. Siriwardane et al.^7^ tested various zeolites for carbon capture from flue gas, e.g., zeolite 13X and UOP-WEG-592, and noted reduction in adsorption capacity in the presence of water vapor. Liquid-impregnated solid sorbents and other surface coatings are among techniques for tuning the hydrophilicity of zeolite 13X that allow an efficient physisorption of CO_2_ via van der Waals forces and hydrogen-bonding mechanisms.^8,9^

The kinetics of adsorption was recurrently found to be dominated by the macropore type of diffusion,^17,18^ fitting well with the Knudsen diffusion model. Silva et al.,^19^ however, have shown some microporous (i.e., inside the crystals)-type relevance of the diffusion model using various geometries of zeolites 13X and ranges of operating temperatures. Unlike the micropore diffusion, the macropore one is known to be sensitive to adsorber geometry and size, pressure drops, and thus overall flow dynamics. The visualization of interaction of flow dynamics and diffusion in the beds would help in understanding overall adsorption kinetics in 13X beads. In fact, the diffusion time (ratio of diffusivity and pore size) is affected by pore size, which is greater in the macropores but also by the length of these, which is smaller in the micropores, leading to competition of the two types of diffusivities based on pellet size and geometry.

The approach proposed herein uses computational fluid dynamics (CFD) for a 3D flow model in a heterogeneous gas–solid system of porous media of assembled particles built by the discrete element method (DEM). The results are assessed by considering the impact of adsorption characteristics on the breakthrough data, resulting from the internal flow dynamics.

## 2 Model Development

### 2.1 Building a Random Packed Bed

**Figure 1:**
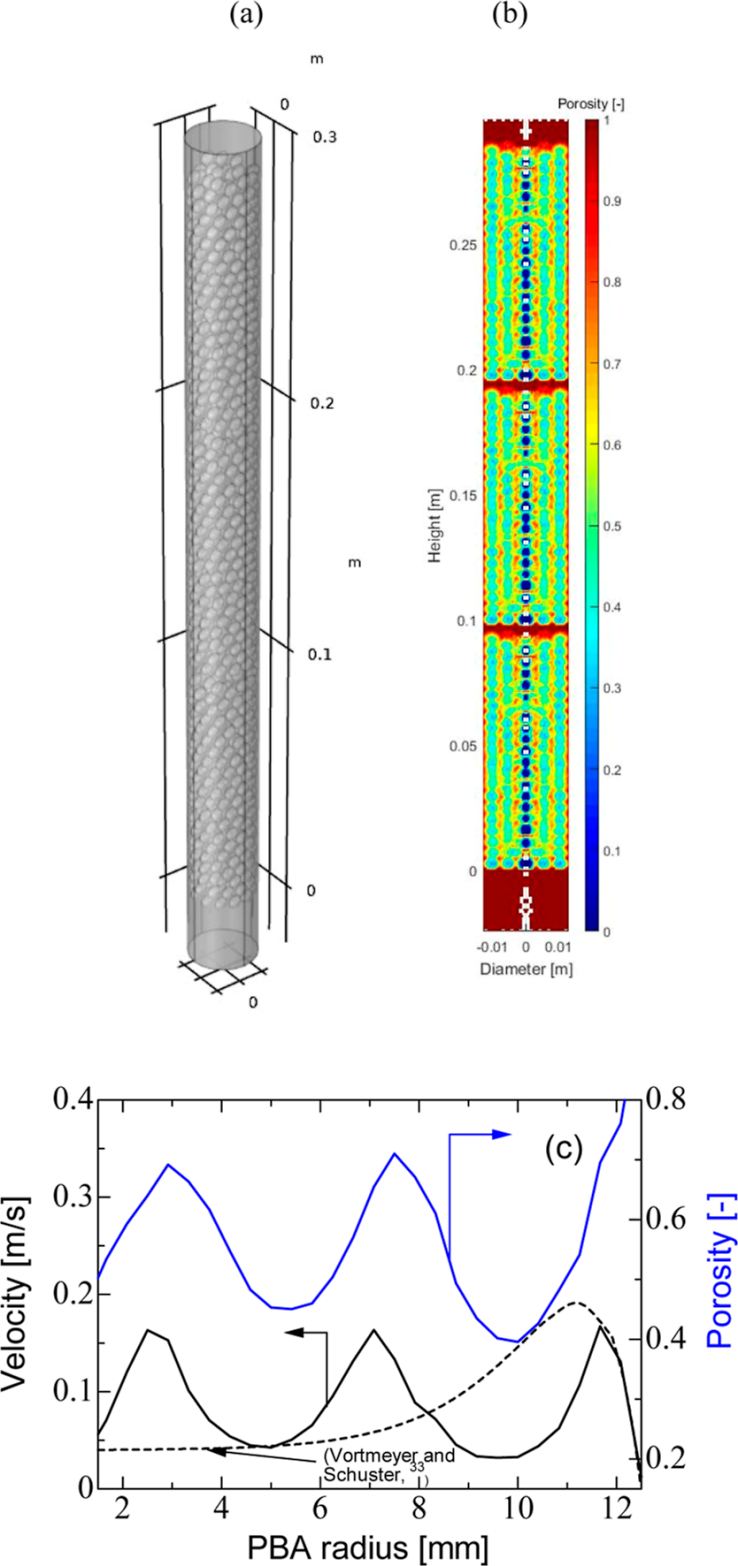
Structural distribution of the packing: (a) DEM built structure; (b) 2D circumferentially averaged porosity; and (c) circumferentially and axially averaged porosity and velocity. PBA of 25 mm ID, 300 mm length, AR of 5, and feed inlet velocity of 0.096 m/s.

The assembly of a typical AR of tube to particle in this study was of 5 and generated 1194 spherical particles using the DEM. The number of particles allowed design of a packing bed of approximately 30 cm height in a cylindrical tube with a 2.5 cm internal diameter (particle diameter size of 0.5 cm). The modeling of particles falling into the cylinder was carried out by using a code written in the embedded FISH programming language of the Particle Flow Code PFC^3D^ (Itasca Ltd.) and more details on building the code are available in previous works.^20,21^ Once the DEM process converged to a point of particle stability, an output 3D matrix was produced that listed a series of particle coordinates. These coordinates were then converted into a CAD format that was compatible with the CFD package, COMSOL Multiphysics’ interface. The geometry in COMSOL could then be observed as a series of suspended spheres, and so a cylinder was built to define the vessel holding the particles. The geometry was then ready for discretization as shown in Figure 1a.

### 2.2 Structure Validation

The relevance of the observation of the 3D structure in comparison with the 2D or 1D structures was assessed through analysis of the bed porosity distribution. The later was then validated by literature models.^22,23^ After the discretization of the volume matrix into 3D unstructured meshes of averaged spatial resolution of at least 25 elements per particle diameter, the size of particles was reduced by about 0.4% to minimize impact of skewed elements at contact points of the particles.^20,21^ The characteristics of the meshes were constantly refined until pressure drops were reached, which were independent of mesh size. The elements of volume, boundary layer thickness at solid to gas, element sizes, and expansion rates were constantly refined until reaching results of pressure drops at a reduced mesh size effect for a reasonable computation time (see discretization assessment in the Supporting Information). A space resolution of 0.5 × 0.5 × 0.5 mm^3^ was found to be satisfactory to observe the variation of local porosity ϕ_3D_ at reduced distortions, particularly in the regions of low meshing resolution.

Thus, the 3D data of the volume matrix were averaged along the angular coordinate θ into a 2D surface bed porosity map ϕ_2D_ (eq 1) and along radial *r* and axial *h* coordinates into overall bed porosity ϕ_ave_ (eqs 2 and 3), allowing access to local data of bed porosity and prediction of flow trends, which otherwise would be challenging to read using the 3D assembly.
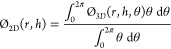
1
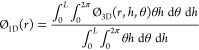
2
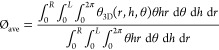
3where *r*, *h*, and θ are the cylindrical coordinates of bed porosity. The bed porosity map in Figure 1b confirms the large bed porosity distribution in the vicinity of the wall, which is a characteristic of packed beds of low AR, and the progressive reduction toward the center of the PBA (Figure 1c). Overall bed porosity ϕ_ave_ showed average deviations of 6.1% from the models.^21^ This deviation could be driven by the management of the skewed contact points of particles. It is interesting to note that the averaged trends of bed porosity did not capture local arrangements of the structure of the PBA along the angular direction θ as exhibited by the 3D structure, demonstrating relevance of the 3D modeling of CO_2_ in comparison with classical D and 2D models.

### 2.3 Fluid Flow Model

#### 2.3.1 Flow Model

The flow inside the packed bed was described by the Navier–Stokes equations for momentum and conservation of mass (eqs 4 and 5). Both pressure and viscous forces were applied to a compressible fluid under a laminar flow. The temperature-dependent physical properties (i.e., density, viscosity, thermal conductivity, and thermal capacity) relevant to both the gaseous and the catalytic phases were obtained from the built-in database of COMSOL or literature as illustrated in Table 1.
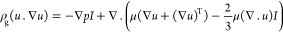
4
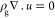
5where ρ_g_ is the density of the gaseous phase, *p* is the static pressure, μ is the dynamic viscosity, *u* is the velocity vector, and *I* denotes the identity matrix. Atmospheric pressure at the exit, fixed velocity at the inlet, and no-slip conditions at the solid–gas contact points were assumed.
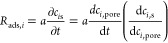
16

**Table 1:** Physical Properties for Adsorbents on Zeolites

physical property	simulation
*C*_*p*_,_zeolite 13X_ [J kg^–^^1^ K^–^^1^]^29^	920
*k*_zeolite 13X_ [W m^–^^1^ K^–^^1^]^39^	0.125
heat capacity of gas, *c*_p_ (J/kg K)^40^	1930
ρ_zeolite 13X_ [kg m^–^^3^]^40^	1930
Δ*H*_ads,CO2/zeolite 13X_ [kJ mol^–^^1^]^29^	36
Δ*H*_ads,N2/zeolite 13X_ [kJ mol^–^^1^]^29^	25
*a* [m^2^/m^3^]^40^	3.95 × 10^6^
*S*_zeolite 13X_ [m^2^ g^–^^1^]^29^	675
*D*_CO_2_–air_ [m^2^ s^–^^1^]^21,36^	1.1 × 10^–7^ *T*(°C) – 1.8 × 10^–^^5^
*D*_N–air_ [m^2^ s^–^^1^]^21,36^	1.5 × 10 −^7^ *T*(°C) – 2.6 × 10^–^^5^
ε_s_ [—]^29^	0.33

Two material balance models were implemented by defining the solid phase and gas phase separately. The gas-phase material balance (eq 6) included the rates for the diffusional and convective transports, while the pores of the solid phase (eq 7) included the rates of diffusional transport and adsorption.
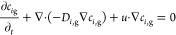
6
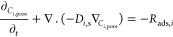
7

The model of diffusivity in the solid phase *D*_*i*,s_ included the textural parameters according to models of nonstructured porous networks and was estimated according to eqs 8–10 by considering both Knudsen *D*_*i*_^K^ and bulk solid types of diffusion *Di*^b^.
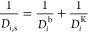
8
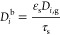
9
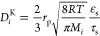
10

The diffusivity values in the gaseous phase *D*_*i*,g_ were approximated to the molecular diffusivity of these species in each other due to the low concentrations used as illustrated in Table 1; *M*_i_ is the molecular weight, *r*_p_ is the average pore radius of zeolite, and ε_s_ and τ_s_ are the textural parameters of particles in terms of bed porosity and tortuosity, respectively.^24,25^

The heat balance model is analogous to the material balance, which includes two equations for the solid and gas phases. There is a convective heat-transfer term in the gas phase and a heat generation term in the solid phase to account for heat of adsorption of CO_2_ and N_2_. eqs 11 and 12 describe the energy balance in the gas and solid phases, respectively.
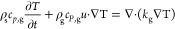
11
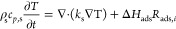
12where c_*p*,g_ and *c*_*p*,s_ are the heat capacity of the gaseous and solid adsorbent phases, respectively, and *k*_g_ and *k*_s_ are the thermal conductivity of the gaseous and solid phases, respectively.

The wall of the PBA was assumed to be exposed to thermal cooling by the environment surrounding the wall of the PBA. The rate of heat loss *Q* is expressed by eq 13, depending on gas–wall or particle–wall contact points.^26^
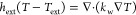
13where *T*_ext_ is the ambient temperature outside of the packed bed, Δ*H*_ads_ is the enthalpy of the reversible adsorption, *h*_ext_ is the heat-transfer coefficient through the wall-surrounding film of the PBA,^27^ and *k*_w_ is the thermal conductivity of the wall.^28^
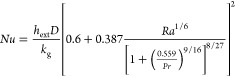
14where *D* is the external wall diameter, *Pr* is the Prandtl number, and *Ra* is Rayleigh number at the average temperature.

#### 2.3.2 Adsorption Model

The 3D model discriminates between the rates of transport and the rate of adsorption. It accounts for loss of species *i* from the gaseous phase in the pores to the surface of the adsorbent, as expressed by eq 15a (a, 15b).
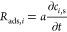
15awhere *c*_s_ is the surface concentration and *a* is the pore surface area to volume ratio (m^2^/m^3^) of the sorbent.
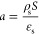
15bSince adsorption of both CO_2_ and N_2_ follows a physical type of adsorption (i.e., Δ*H*_ads,CO_2_/zeolite 13X_ and Δ*H*_ads,N_2_/zeolite 13X_ are 36 and 25 kJ mol^–1^, respectively, as illustrated in Table 1), the kinetics of surface adsorption is considered fast to reach an equilibrium state.^3^ By applying the chain rule to dependent variable *c*_*i*,pore_, the rate of surface adsorption becomes proportional to that in the pores.The term  is the slope of the Langmuir isotherm model reported suitable for the weak interactions between N_2_ and CO_2_ at a CO_2_ composition of less than 20%.^29−31^ The ideal adsorption solution theory then applies to the quantity adsorbed, as expressed by eq 17.
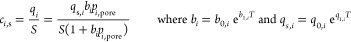
17where *q*_s_ is the adsorbed quantity, *S* is surface area per unit mass of adsorbent, and *p*_i_ is the partial pressure of component *i*. Adsorption constants *q*_0_, *q*_1_, *b*_0_, and *b*_1_ for CO_2_ and N_2_ adsorption on zeolite 13X are illustrated in Table 2.

**Table 2:** Adsorption Isotherm Parameters for the Langmuir Isotherm Model^29^

constants^17^	zeolite 13X	
	CO_2_	N_2_
*q*_0,*i*_ [mol kg^–^^1^ Pa^–^^1^]	2.38341	0.06355
*q*_1,*i*_ [K^–1^]	–0.02816	–0.02934
*b*_0,*i*_ [Pa^–1^]	0.12266	6.313 × 10^–^^4^
*b*_1,*i*_ [K^–1^]	–0.02353	–0.01419

17 .

After differentiation of eq 17 with respect to *c*_i,pore_ and substitution in eq 16.
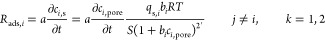
18which then leads the models of mass and heat balance in the pores (eqs 7 and 12) to become a function of concentration of species in the pore phase only, as expressed by eqs 19 and 22, respectively.
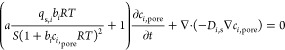
19
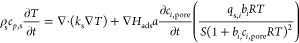
20
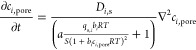
21
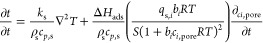
22where  and  are the effective diffusion and packing Henry law constants, respectively. Before simulations could be initiated, the boundary conditions were set [i.e., an initial concentration of 0.001% of CO_2_ in the carrier gas N_2_, atmospheric pressure at the exit, uniform velocity and temperature across the inlet plane, and no-slip condition at all solid boundaries (i.e., particle and wall)]. The fluid flow equations were first solved in a steady-state condition prior to solving the time-dependent equations, allowing action as an initial boundary condition for the dynamic terms (mass, heat, and adsorption). The integration of the governing equations was carried out by discretization into finite elements by using sets of difference equations. The generalized minimal residual method with the Geometric Multigrid preconditioner algorithm was used to approximate the solution at minimum residual values. The built-in meshing module of COMSOL, which includes an adaptative mesh refinement procedure, allowed investigation of the effect of the size of the elements on the fluid flow model and validation by a mesh convergence test. The pressure values at three locations were assessed by using the Grid Convergence Index (GCI). Errors due the discretization procedure were assessed by following the procedure recommended by Celik et al.,^32^ validating that the results were not affected by mesh size. A solution, irrespective of mesh size, was reached when the GCI was below 3.1%. The simulation was carried out using COMSOL Multiphysics 5.3 and a single 512 GB RAM computer server by Dell incorporation, which was equipped with Intel Xeon E5-2637 v3 specification and 4 cores 3.50 GHz.

## 3 Results and Discussion

### 3.1 Velocity Profiles inside the Packed Bed

The structure of the packing impacted the way in which the flow of CO_2_ passed through the void regions of the packing, driving variation in velocity and thus convective transfers of mass and heat, depending on the local topology. Since a low-AR packing (i.e., AR 5) was used, it introduced the effect of the wall on the structure of the packing as reported in Section 2.2 and additional impact on adsorption caused by the channeling at the outer regions of the bed.^21^

**Figure 2:**
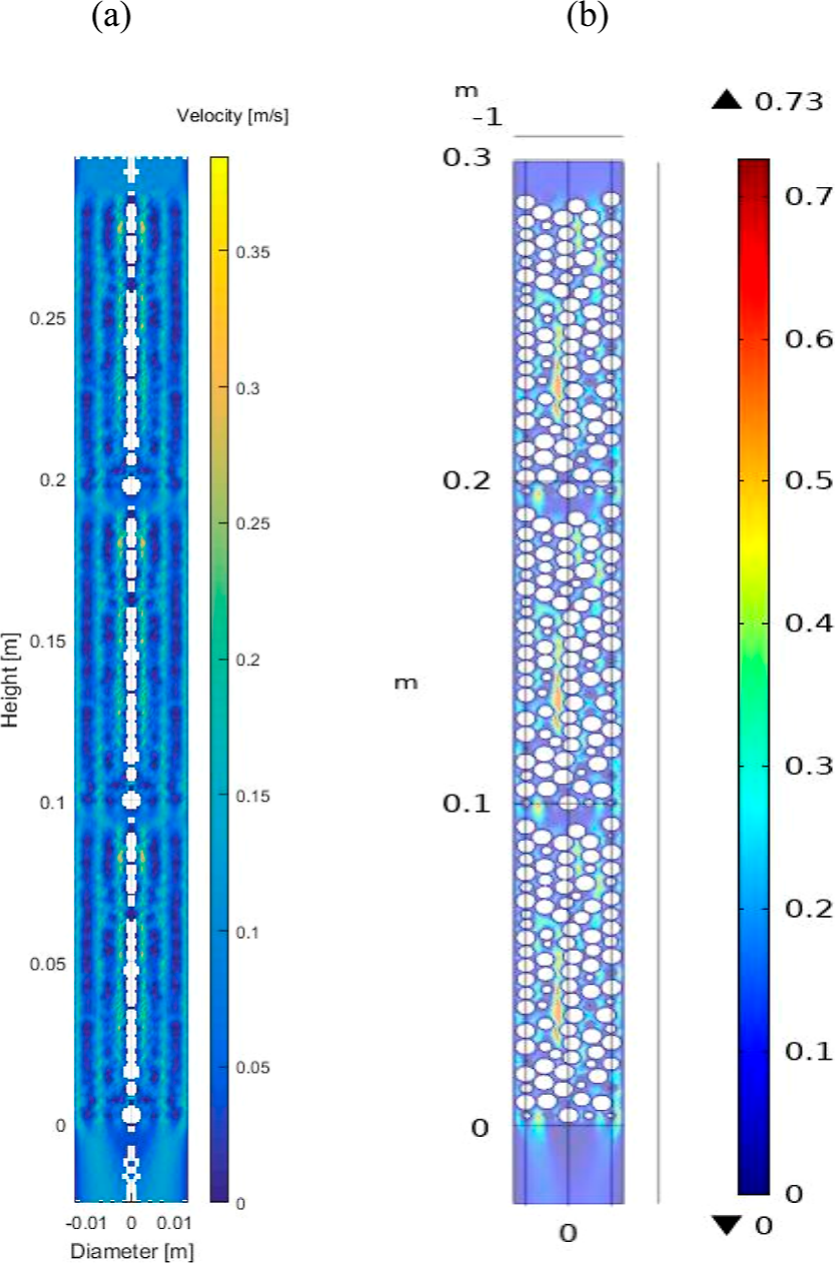
Interstitial velocity map. (a) Circumferentially averaged velocity and (b) axial section of the PBA. PBA of 25 mm ID, 300 mm length, AR of 5, and feed inlet velocity of 0.096 m/s.

Figures 1c and 2a,b show profiles of circumferentially and axially averaged velocity, circumferentially averaged velocity, and cross-axial velocity, respectively, along the PBA when it was operated at an inlet velocity of 0.096 m s^–1^^29^ The results were compared with the model of Vortmeyer and Schuster^33^ who solved the modified Brinkman equation for fluid flow and accounted for the walls’ presence. An increase in velocity near the wall is observed due to the preferential channeling effect before it subsided due to the no slip at the solid boundaries. Local circumferentially averaged velocities in Figure 2a are seen to be nonuniform and a function of the local bed porosity and structure of the packing. Local peaks of interstitial velocity, as observed in the axial section of the bed in Figure 2b, have reached values of about 7 times higher than the average velocity in certain zones where more efficient mixing (Figure 2b), and by inference mass and heat transports, is anticipated.

### 3.2 Analysis of Adsorption inside the Packed Bed

The effect of thermal exchange with the surrounding was studied to explore how breakthrough was affected by operations at adiabatic and nonadiabatic conditions, again by gaining access to the inside of the bed.

**Figure 3:**
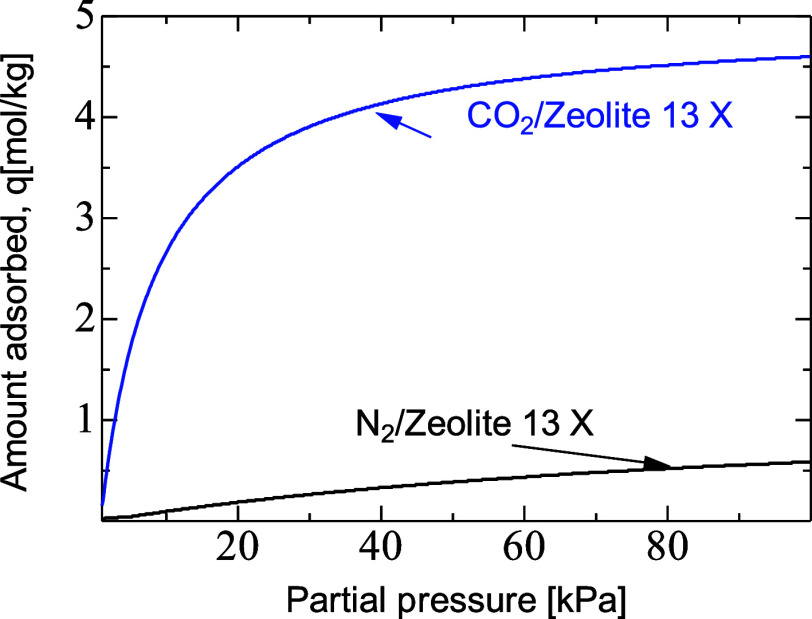
Adsorption isotherms of CO_2_ and N_2_/zeolite 13X at 20 °C.^29^

**Figure 4:**
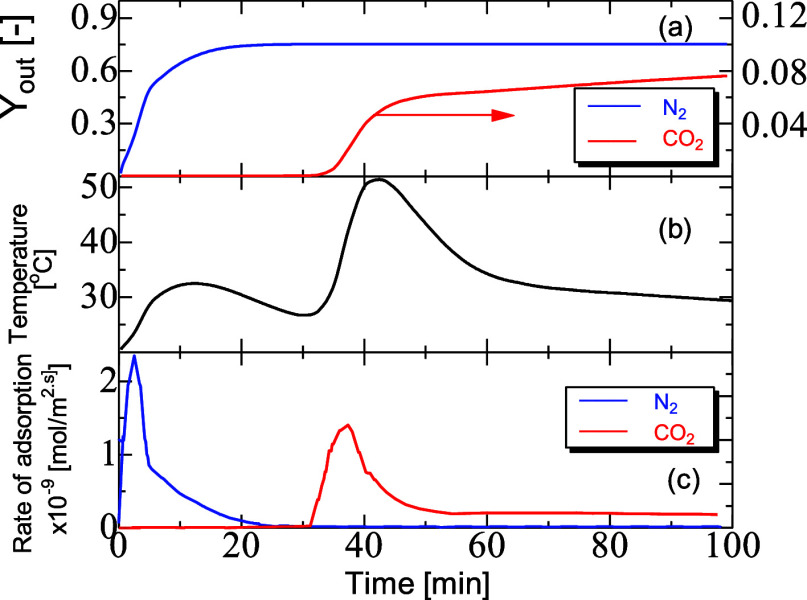
Breakthrough mole fraction (*Y*_out_) (a), temperature (b), and adsorption rate (c) profiles for the dual adsorption of CO_2_ and N_2_. PBA of 25 mm ID, 300 mm length, diffusivity of 10^–8^ m^2^/s, AR of 5, feed inlet velocity of 0.096 m, inlet temperature of 20 °C, and *h*_w_ of 9.5 W/m^2^ K.

In the case of dual adsorption, the governing equations were set up in the same manner for both CO_2_ and nitrogen gases and inlet feed CO_2_ mole fraction gas in N_2_ was set to 15%. Figure 3 shows the isotherms of CO_2_ and N_2_ on zeolite 13X at 20 °C and 15% CO_2_ mole fraction, where the capacity for CO_2_ was far greater than for N_2_.^29^ The adsorption isotherm of the binary mixture followed the ideal adsorbed solution theory model, and nitrogen was hardly adsorbed when its mole fraction was less than 0.8 but importantly did not affect the equilibrium amount of CO_2_, validating application of ideal adsorption solution theory. The isosteric heat of adsorption for CO_2_ and N_2_ was set to 36 and 25 kJ, respectively, which indicates a stronger affinity to CO_2_.^34^ It can be seen in Figure 4a with the N_2_ component that the breakthrough occurred as soon as the transient process began. There were two processes driving this early breakthrough; first, the adsorption process was not able to sufficiently store N_2_ on the solid surface because of slow kinetics (low gradient) and low capacity (small plateau). Second, the competition existed between the CO_2_ and N_2_, and because CO_2_ had higher kinetics and capacity, it saw higher selectivity for storage on the solid surface. This is supported by the fact that higher energy was released for CO_2_ adsorption. The exit temperature for the dual adsorption is shown in Figure 4b, where the first breakthrough peak of temperature was for N_2_ but with lower values of heat of adsorption and adsorption capacity.

The average rate of adsorption occurring in the particles located at the exit of the packed bed could be taken over the transient interval for both N_2_ and CO_2_, along with the average temperature, as seen in Figure 4c. The first temperature peak was accompanied by an increased peak rate of N_2_ adsorption, and the second was accompanied by a large peak rate of CO_2_ adsorption. The rate of the peaks, denoted by , validates the relationship between temperature of adsorption and the rate of mass transfer in the solid phase. The rate peak of N_2_ was higher than that of CO_2_ while the amount of N_2_ transferred to the solid phase was significantly lower, and so the overall heat released was less. Besides this, the mole fraction of N_2_ in the gas phase was much higher than that of CO_2_, and so, the initial driving force was large. It is observed that when large breakthrough waves [larger mass-transfer zones (MTZs)] were present, the leading concentration was lower, which also made the rate lower, as seen in the comparison of solid diffusivity (where lower diffusivities led greater temperature peaks).

**Figure 5:**
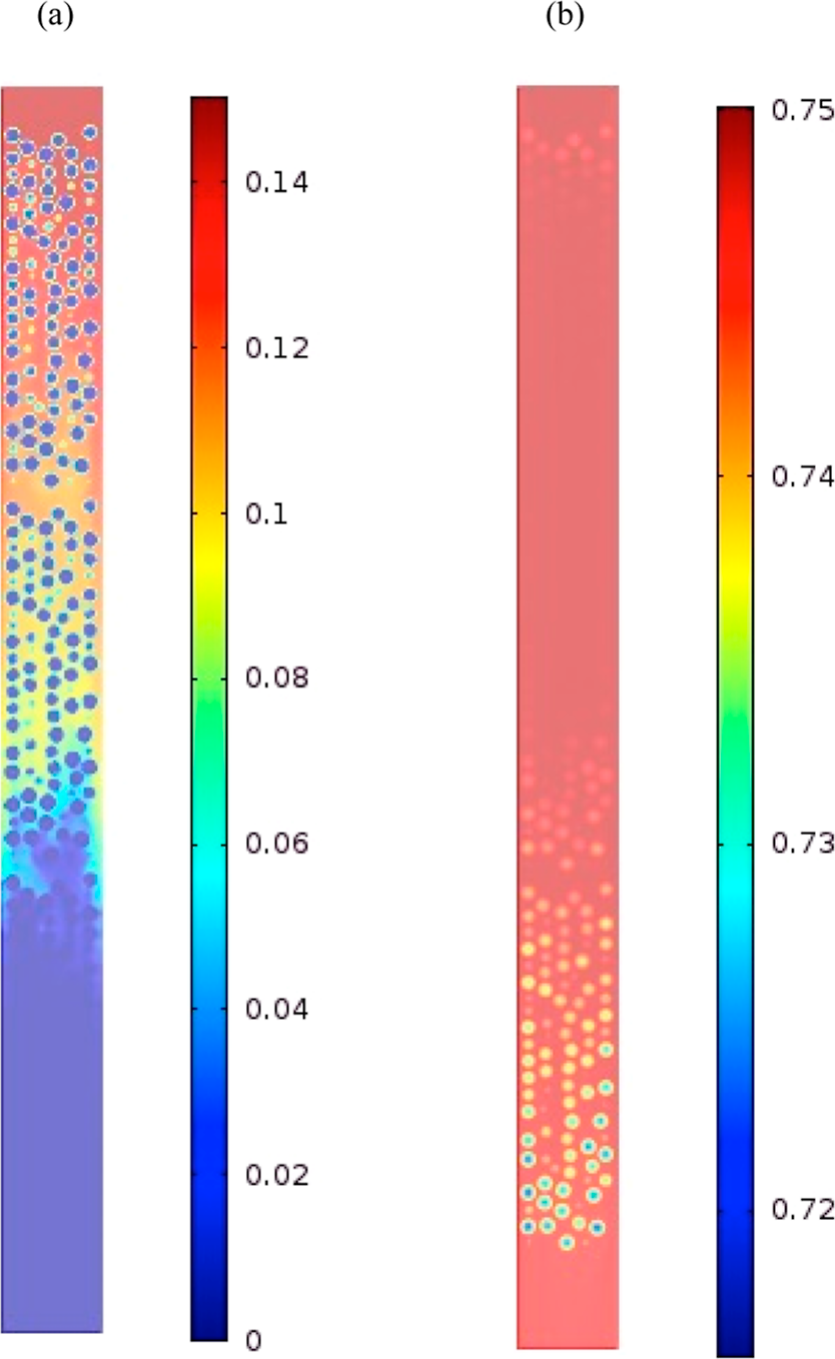
Axial section of mole fraction of CO_2_ (a) and N_2_ (b) after 25 min, inlet mole fraction of CO_2_*Y*_CO2_ = 0.15 and *Y*_N_2__ = 0.75. PBA of 25 mm ID, 300 mm length, diffusivity of 10^–8^ m^2^/s, AR of 5, feed inlet velocity of 0.096 m, inlet temperature of 20 °C, and *h*_w_ of 9.5 W/m^2^ K.

Figure 5 shows axial sections of the mole fraction profile inside the adsorber after 25 min of operation. By this point, N_2_ had already broken through, and its uptake had virtually ceased. What is noticeable is the particle saturation level of both solutes. For N_2_, the solid phase was almost completely saturated, with a penetration reaching the center of the particle. For CO_2_, the saturation had occurred only near the edge. This suggests that the uptake was still ongoing in the central regions of the particles and that CO_2_ was still being transferred to the adsorbed phase. This ongoing adsorption is supported by the presence of the breakthrough tailing.

#### 3.2.1 Effects of Internal Mass Transfer on Adsorption Profiles

The diffusivity of CO_2_ inside porous solids is a function of the pore network at various scales (i.e., micro- to macropores) within zeolite and presence of N_2_.^35^ It is therefore important to gain a perspective into the effect of the diffusion magnitude, as this represents different materials and can vary by orders of magnitude for a single material. According to eqs 6 –8, the diffusion of CO_2_ inside zeolite 13X may vary between 10^–6^ (macroporous) and 10^–8^ (microporous) m^2^/s, depending on the structure of the pores.^36^ This variation in porosity has been investigated by surface coating (i.e., tuning the hydrophilic property for the reduction of water vapor inhibition, chemical impregnation, and ion exchange for the promotion of CO_2_ intake^7−9^).

**Figure 6:**
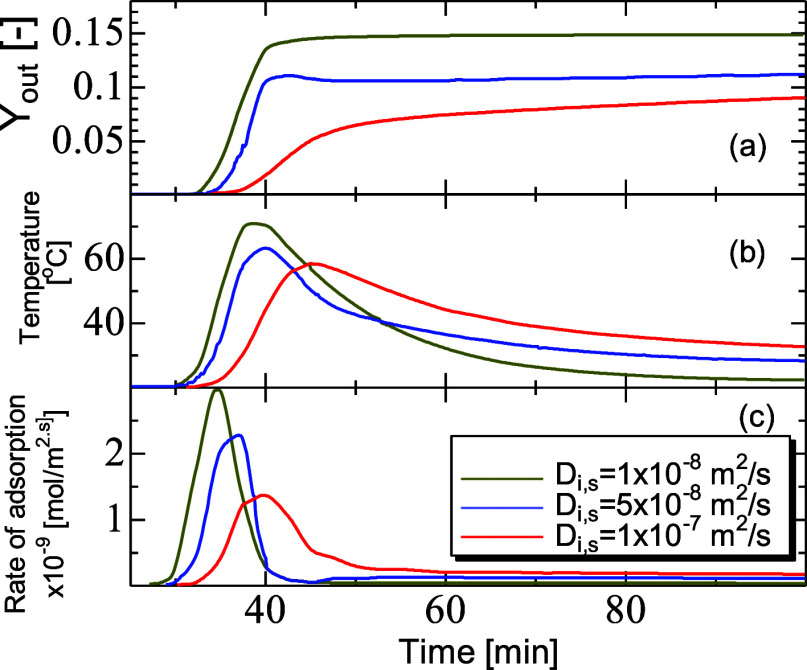
Breakthrough mole fraction (*Y*_out_) (a), temperature (b), and adsorption rate (c) profiles for CO_2_ adsorption on zeolite 13X. Diffusivity values ranging from 1 × 10^–7^ to 1 × 10^–8^ m^2^/s, and PBA of 25 mm ID, 300 mm length, AR of 5, feed inlet velocity of 0.096 m, inlet mole fraction of 0.15, inlet temperature of 20 °C, and *h*_w_ of 9.5W/m^2^ K.

Figure 6a–c shows the breakthrough in terms of mole fraction, temperature, and adsorption rate profiles at the exit for diffusivity values ranging from 10^–8^ to 10^–7^ m^2^/s (i.e., *r*_p_ from 2 to 5 nm). The curves in Figure 6a show a steep initial shape before leveling off. The onset of concentration breakthrough occurred earlier for the case of low diffusivity due to the reduced mobility of CO_2_ and breakthrough. The gradient of the concentration breakthrough curve was a function of the MTZ that is undergoing dynamic adsorption and reflected by an observable wave moving through the bed. Wide MTZs associated with a pronounced tailing in the breakthrough curves were present for large diffusivity (i.e., *D*_i,s_ of 1 × 10^–7^ m^2^/s). The tail shows a gradual increase in outlet concentration, while for reduced diffusion rate, the plateau front progressed toward a flat shape. When the diffusivity was set low, however, undesirable features such as little forewarning on the onset of breakthrough that it was beginning and resulted in high concentrations of CO_2_ at the outlet. The wide MTZ and breakthrough tail occurred as a result of the prolonged adsorption kinetics driven by diffusion of CO_2_ further into the solid particles. Large MTZs and breakthrough curve tailings are undesirable for process operations as they lead to low utilization of the solid where the shallow curve is. For the case of higher diffusivity, a double breakthrough event occurred. The double breakthrough event is the result of the breakthrough wave interference of heat and mass when they travel along the packed bed and lead to either overshoot or undershoot values in amplitudes of the concentration and temperature along the bed length. The first was associated with an initial steeper increase in concentration at the outlet, and the second was associated with a shallower tailing of the curve, and later associated with the catching up of the breakthrough wave as a complete breakthrough occurred. The catch-up behavior at high diffusivities was more pronounced as the uptake was prolonged by the steeper loading at the wall due to channeling. This behavior led to elevated temperature and reduced uptake which was followed by a reduced temperature due to the convective cooling and heat losses to the surrounding, once the first breakthrough has occurred.

**Figure 7:**
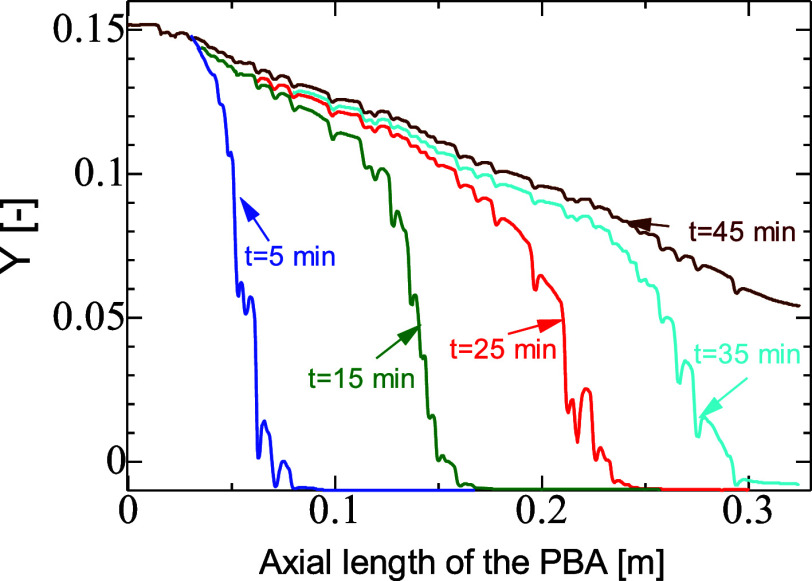
Transient profiles of axial mole fraction *Y* [—] along the PBA. PBA of 25 mm ID, 300 mm length, AR of 5, feed inlet velocity of 0.096 m, diffusivity of CO_2_ of 1 × 10^–7^ m^2^/s, inlet mole fraction of CO_2_ of 0.15, inlet temperature of 20 °C, and *h*_w_ of 9.5 W/m^2^K.

**Figure 8:**
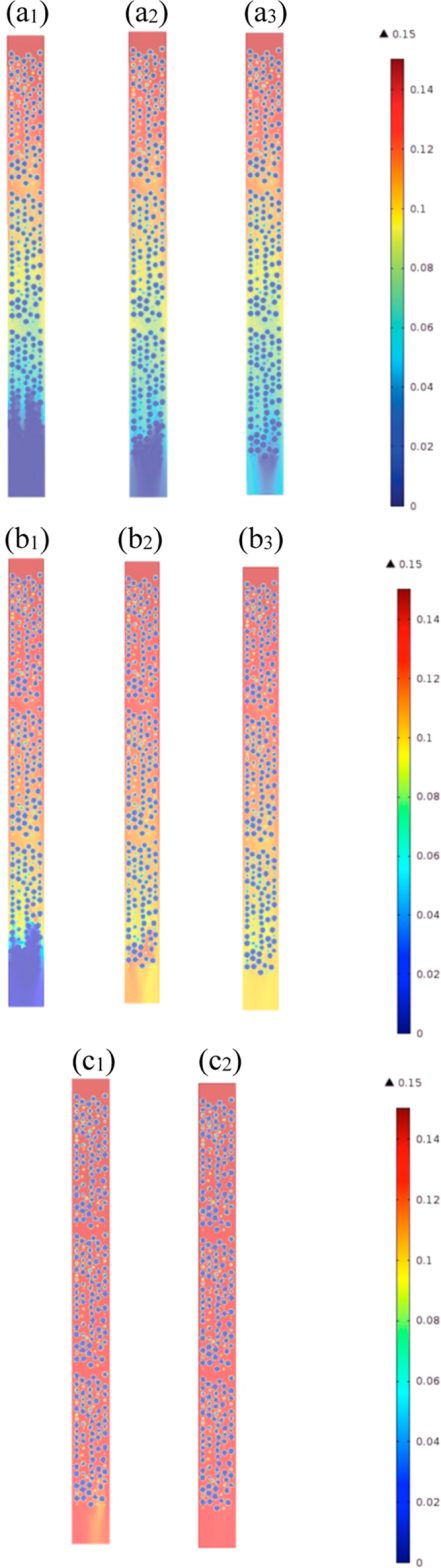
Axial sections of the mole fraction (*Y*) of CO_2_ along the PBA. (a–c) for *D*_*i*,s_ = 10^–7^, 5 × 10^–8^, and 10^–8^ m^2^/s, respectively, and subscripts 1, 2, and 3 for *t* = 35, 40, and 45 min, respectively, and PBA of 25 mm ID, 300 mm length, AR of 5, feed inlet velocity of 0.096 m, inlet mole fraction of CO_2_ of 0.15, inlet temperature of 20 °C, and *h*_w_ of 9.5 W/m^2^ K.

Figure 7 shows axial profiles of CO_2_ inside the gas and solid phases of the PBA over the 1D representation. The breakthrough wave was initially curved and became more linear as time went on. There are no discontinuities in the lines covering both phases, underlying low resistance to mass transfer in the area surrounding the solid particles. The peaks of concentration drops in the pores of the particles are visible along the bed length but particularly at the front end. The 1D representation does not offer sufficient details on the phenomena that are taking place in the PBA and therefore the 2D maps in Figure 8 provide wider representations of the breakthrough dynamics, including along the radial distribution at three values of diffusivities (i.e., *D*_*i*,s_ = 10^–7^, 5 × 10^–8^, and 10^–8^ m^2^/s), the point just before the exit breakthrough begins (for *t* = 35 min), the point at which breakthrough is occurring (for *t* = 40 min), and the point at which the plateau is reached (for *t* = 45 min). There is a noticeable width of the MTZ for the highest diffusivity value (*D*_*i*,s_ = 10^–8^ m^2^/s), which covers well over two-thirds of the bed length at 35 min. For *D*_*i*,s_ = 1 × 10^–8^ m^2^/s, there is a narrow MTZ, that results in the steep breakthrough curve seen in Figure 6. Wide MTZs cause pronounced tailing in the breakthrough curves that are especially present in the case of *D* = 1 × 10^–7^ m^2^/s. The tail shows a gradual increase in outlet concentration, while for a reduced diffusion rate, the plateau front is very flat. In an application sense, larger MTZs and breakthrough curve tailing are undesirable for process operation, since there is lower utilization of the solid and a wide range of low utility where the shallow curve is.

For steep breakthroughs, as illustrated by axial mole fraction maps of CO_2_ at various values of diffusivity and adsorption time in Figure 8, a narrow MTZ existed, and when the gradient was shallower, a wider MTZ was developed. Figure 8a_1_,b_1_,c_1_,a_2_,b_2_,c_2_,a_3_,b_3_ illustrates mole fraction maps at the point just before breakthrough began, the point at which breakthrough was occurring, and the point at which the plateau was reached. It is apparent that for the highest diffusivity, there was a noticeable development of the width of the MTZ, which covered well over two-thirds of the bed length at 35 min. For *D* = 1 × 10^–8^ m^2^/s, there was a narrow width of the MTZ, that resulted in the steep breakthrough curve seen in Figure 8.

Interestingly, these figures show that the bulk cross section of the gaseous phase was more saturated with CO_2_. It should be noted that, unlike the mass-transfer resistance, the heat-transfer resistance inside the particles was negligible (i.e., low values of Biot and Stanton numbers) due to reduced gradients of temperature inside the particles. The trends of this saturation followed the velocity map cross section and thus demonstrated the nonrelevance of external mass transfer around the particles. Inside the particles, however, gradients of concentrations are observed and by inference, the relevance of the inner mass-transfer resistance. The range of inner saturation varied from a fully depleted area at the center of particles to progressive depletion at the periphery of the particles. At the interparticle scale, the concentrations of CO_2_ are higher at the center of the PBA than the periphery regions and these concentrations followed well the trends of concentrations in the bulk gaseous phase. These gradients inside the particles increased as the particles were filled with CO_2_. Overall, at low diffusivity, the profiles of mole fraction maps inside the particles were symmetric and thus were less affected by the surrounding bulk phase. The diffusion rate was the same, despite the uneven mass-transfer rate around the particles. At high values of diffusivity, the concentration inside the particle was, however, affected by the particle surroundings observed by the angular asymmetry.

##### 3.2.1.1 Temperature Profiles

The mole fraction breakthrough curves for the three diffusion rates were accompanied by the nonisothermal temperature curves as shown in Figure 6b. These are complementary but validate once more that the onset of breakthrough occurred later in the case of increased diffusivity, as mapped by the raised peaks of temperature.

The profiles of temperature show adsorption zones which are located between the onset temperature and the maximum temperature. At the front of the adsorption zone, the temperature increased to the highest values, whereas the downstream zones exhibited high temperature waves before decreasing due to cooling by the flow dispersion and the wall thermal exchange. For lower diffusion rates as shown in Figure 6c, the peaks of temperature were higher, driven by a high rate of adsorption and caused by the much smaller adsorption front for the moving breakthrough wave. For higher diffusivity, the adsorption occurred for a wider range of the bed, and since the concentration was lower at the front of the wave, the rate was also slower. Furthermore, the decay of the peak of temperature at high diffusivity was slower, again due to the prolonged adsorption in place that continued to generate heat, compared to diffusion where adsorption subsided after breakthrough and inlet temperature was reached faster. The peaks of temperature were also narrower for slower diffusion and were a function of the width of the breakthrough front. Since increased access to the particles was available, more material was adsorbed and the heat generated lasted for a longer period.

**Figure 9:**
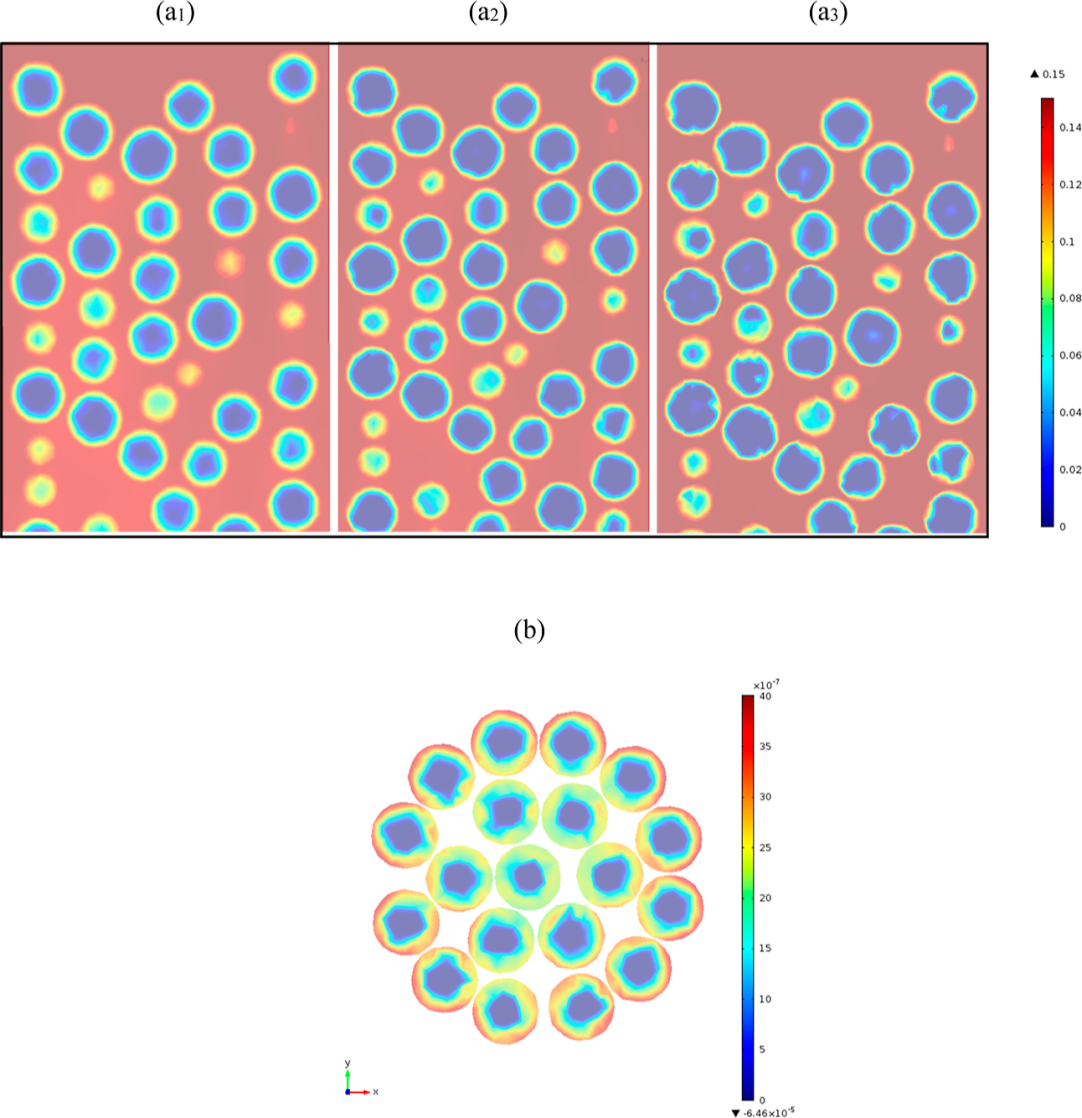
Axial section maps of CO_2_ mole fraction (*Y*) for (a_1_) *D*_*i*,s_ = 1 × 10^–7^, (a_2_) 5 × 10^–8^, and (a_3_) 10^–8^ m^2^/s, and the cross-sectional mole fraction map of CO_2_ (b), time of 100 min, PBA of 25 mm ID, 300 mm length, AR of 5, feed inlet velocity of 0.096 m, inlet mole fraction of CO_2_ of 0.15, inlet temperature of 20 °C, and *h*_w_ of 9.5 W/m^2^ K.

Axial sections of CO_2_ mole fraction inside the PBA for each diffusion rate and after 100 min are shown in Figure 9a_1_–a_3_. For *D* = 1 × 10^–7^ m^2^/s, there was an increased depth of penetration, which backs up the previous statement that greater utilization of the solid phase has been achieved. Particle utilization was, however, not high, since after a 100 min period, the central regions in the particles were far from being saturated with CO_2_, which is weakly visible near the center of the particles. The isotherm of CO_2_ onto zeolite 13X in Figure 3 is steep, which resulted in faster adsorption kinetics, causing the core regions of the particles to be devoid of CO_2_ while it was still being captured from the gaseous phase of the pores. Once the outer regions of the particle were saturated in CO_2_, the capacity in adsorption of CO_2_ by zeolites would bepromoted, and so the pore gas mole fraction began to increase.

##### 3.2.1.2 Angular Nonsymmetric Distribution of Mole Fraction and Temperature

When 1D and 2D pseudo-homogeneous modeling methods are used, there are limited means of seeing the process occurring at the local scale during its operation. In this case, adsorption uptake has been shown to vary depending on the interstitial flow and structure inside the bed. The adsorption process occurring inside the packed bed did not show an entire homogeneity. Figure 9b shows that adsorption occurred earlier at the wall region of the column, which stemmed from the structure of the packing discussed in Section 2.1 (i.e., an increased bed porosity near the wall). The result of nonuniformity of the structure is shown by increased concentrations and in agreement with the known literature on the channeling phenomena in the vicinity of the wall of low-AR beds,^11,37^ driven by motion of fluid into the outer porous regions due to larger flow areas. Overall, the lack of angular symmetry inside the particles is attributable to reduction in resistance to mass transfer in the area of solid particles exposed to high velocities, and so, there were greater convective transports from the bulk of the gaseous phase to the pores—as illustrated by the lower temperature trends near the wall.

#### 3.2.2 Nonuniform Radial Distribution of CO_2_ Mole Fraction and Temperature

**Figure 10:**
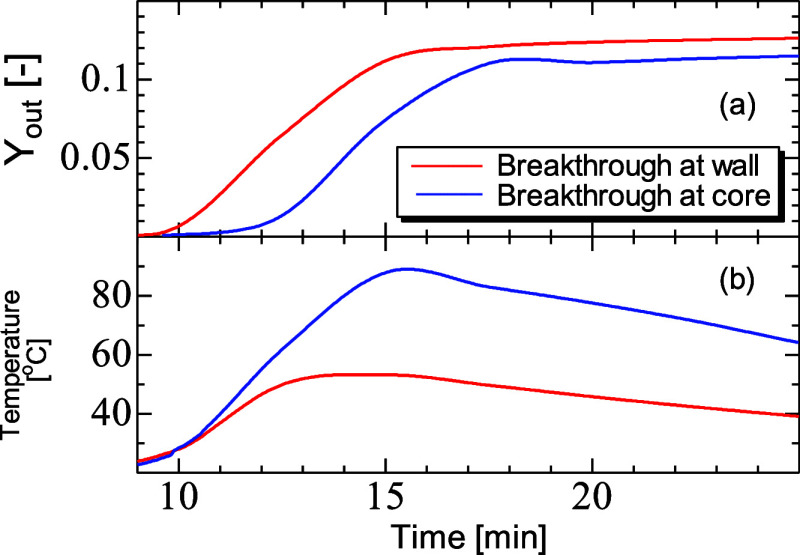
Breakthrough mole fraction (*Y*_out_) (a) and temperature (b) profiles for CO_2_ adsorption on zeolite 13X at the core and wall regions of the PBA. PBA of 25 mm ID, 300 mm length, *D*_*i*,s_ = 1 × 10^–7^ m^2^/s, AR of 5, feed inlet velocity of 0.096 m, inlet mole fraction of CO_2_ of 0.15, inlet temperature of 20 °C, and *h*_w_ of 9.5 W/m^2^ K.

The breakthrough trends were later observed by taking transient data of the mole fraction and temperature over the adsorption period at single points inside the bed. Figure 10a,b shows both the mole fraction and temperature breakthroughs, respectively, for a diffusivity of 10^–7^ m^2^/s at the wall and core of the PBA. The points were taken at a point midway through the column in the void space between particles. The wall region displayed an earlier onset of breakthrough because there was a reduced amount of adsorbent than in the more central regions. Besides this, the plateau concentration of the wall breakthrough was larger than in the core for the same reason.

**Figure 11:**
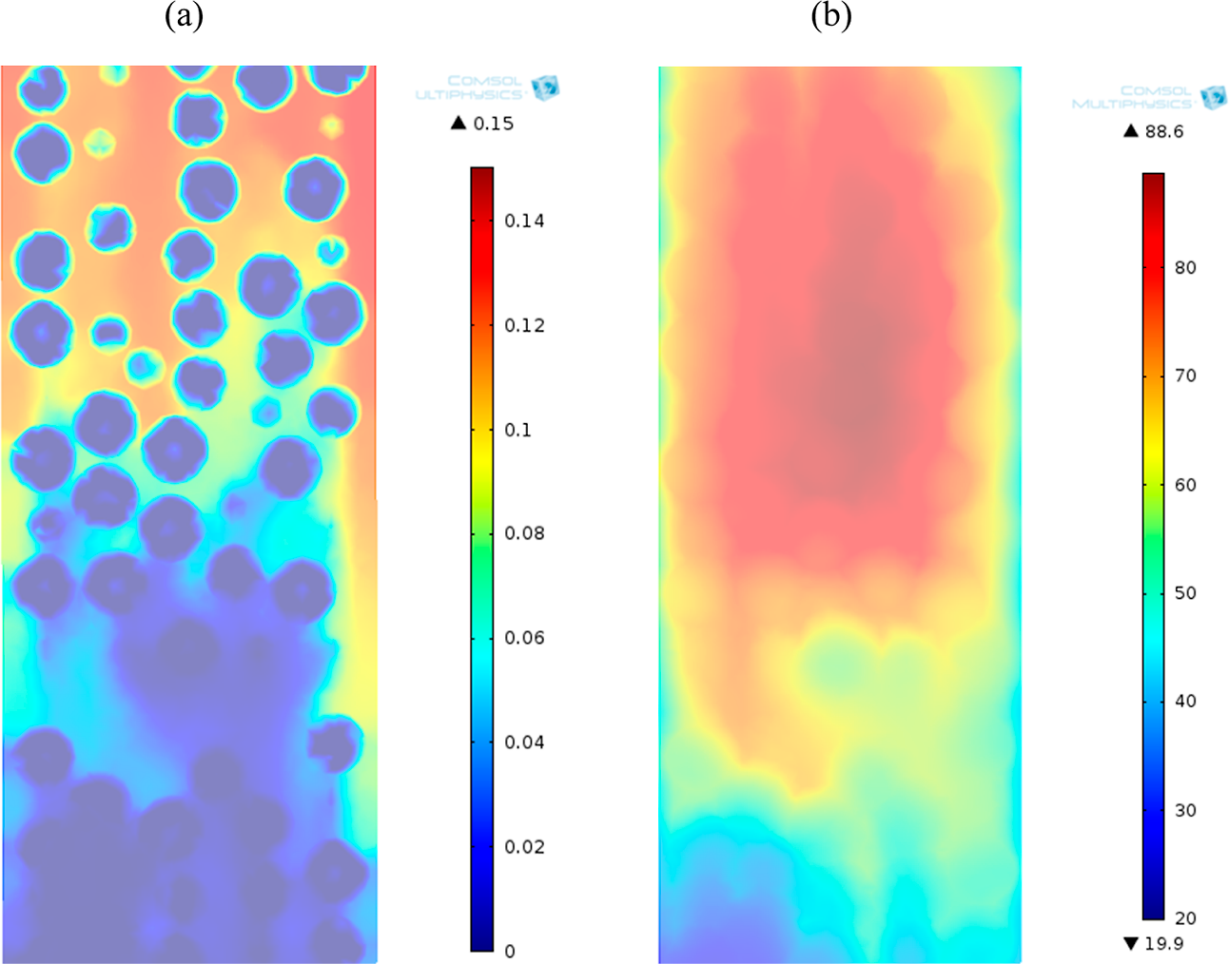
Axial sections of (a) mole fraction and (b) temperature for the adsorption of CO_2_ on zeolite 13X. PBA of 25 mm ID, 300 mm length, *D*_*i*,s_ = 1 × 10^–7^ m^2^/s, AR of 5, time of 25 min, feed inlet velocity of 0.096 m, inlet mole fraction of CO_2_ of 0.15, inlet temperature of 20 °C, and *h*_w_ of 9.5 W/m^2^ K.

The relevant temperature curves in Figure 10b show analogies to those of the mole fraction. Higher temperatures are observed in the core of the bed because there was a reduced ability to convey heat away from the point of generation, despite the adsorption rate being greater in the wall region. At the wall, the gas flow rate was higher and so the heat has been rapidly dispersed by convection or exchanged through the wall. Figure 11a,b shows axial sections along the bed of mole fraction of CO_2_ and temperature at the breakthrough front and relevant dynamic adsorption for the entire breakthrough time is illustrated in movie files, Movies 1 and 2, respectively, in the Supporting Information. Once more, higher mole fractions and earlier breakthroughs can be seen at the wall. Temperature in the core of the bed was the highest and then diminished toward the wall.

#### 3.2.3 Catch-Up Behavior

The concentration near the wall of the bed has been shown to experience breakthrough faster than in the core due to the channeling effect and increased loading at the wall. Besides this, there was another occurrence taking place. In the core, the temperature was elevated to those at the wall, and so the equilibrium capacity was reduced, and less uptake occurred on the solid surface. Once the breakthrough occurred, temperature began to decline as the heat was lost through the wall and the convective flow through the PBA. This then means that the equilibrium capacity of the core bed increased, and more adsorptions could take place; hence, the outlet mole fraction decreased slightly as more material started to transfer to the solid phase. This effect was much more profound in the core and was far more visible than at the wall (see Figure 10a). Salem et al.^38^ discussed similar trends in the 2D modeling of adsorption of water vapor, noting a similar lag in the core as adsorption uptake increased following a decline in temperature.

#### 3.2.4 Effect of Thermal Exchange with the Surrounding on the Breakthrough Profiles

**Figure 12:**
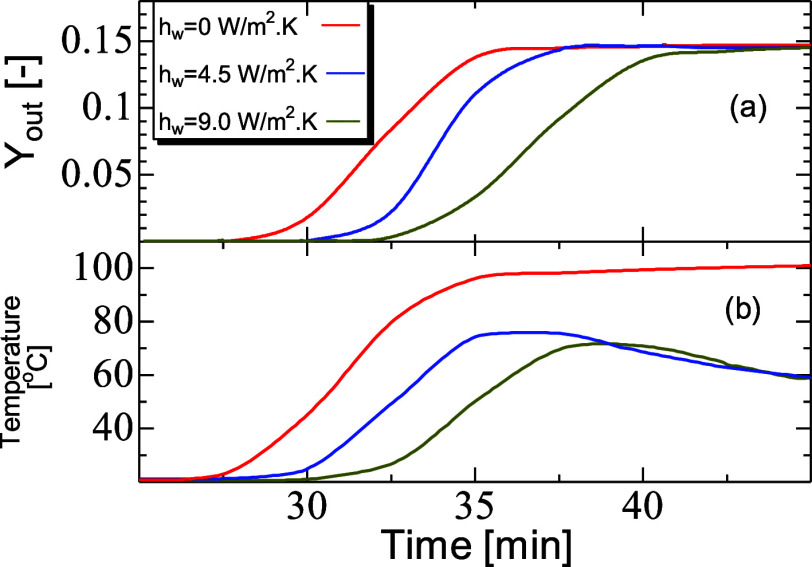
Breakthrough mole fraction (*Y*_out_) (a) and temperature (b) profiles for CO_2_ as a function of thermal exchange with the surrounding. PBA of 25 mm ID, 300 mm length, *D*_*i*,s_ = 1 × 10^–7^ m^2^/s, AR of 5, feed inlet velocity of 0.096 m, inlet mole fraction of CO_2_ of 0.15, and inlet temperature of 20 °C.

**Figure 13:**
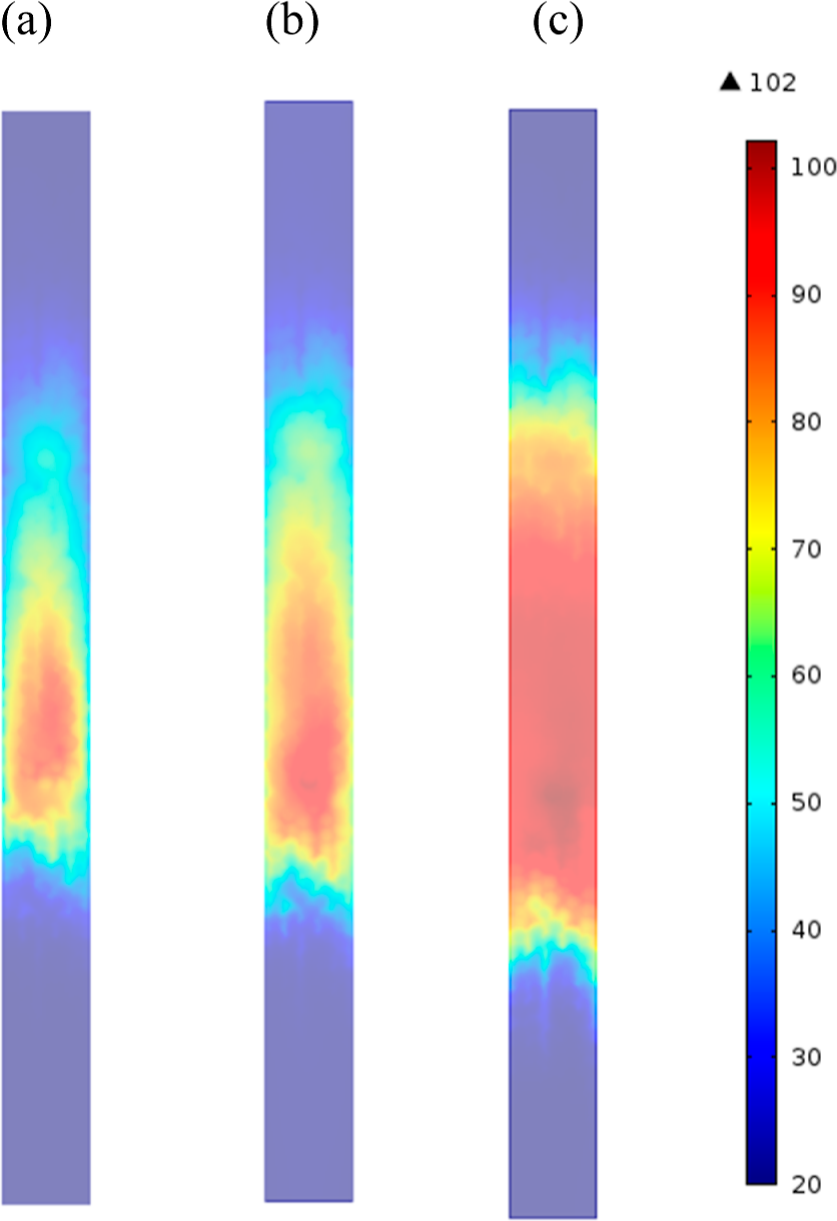
Axial sections of temperature for CO_2_ adsorption on zeolite 13X along the PBA at *t* = 25 min for (a) *h*_w_ = 9.5 W/m^2^ K, (b) *h*_w_ = 4.5 W/m^2^ K, and (c) *h*_w_ = 0 W/m^2^ K (right). PBA of 25 mm ID, 300 mm length, *D*_*i*,s_ = 1 × 10^–7^ m^2^/s, AR of 5, time of 25 min, feed inlet velocity of 0.096 m, inlet mole fraction of CO_2_ of 0.15, and inlet temperature of 20 °C.

The impact of rate of thermal exchange between the PBA and the surrounding on the breakthrough behavior was investigated by varying the wall-surrounding heat-transfer coefficient *h*_w_, as a flexible characteristic (i.e., subject to size, geometry, and position of the PBA and to flow in the surrounding^21,29^). The transient adsorption process was operated with values of column wall heat-transfer coefficients of 9.5, 4.5, and 0 W/m^2^ K, which correspond to a typical range of thermal exchange with an ambient surrounding^26^ and included the full thermal insulation of the PBA. Figure 12a,b shows the mole fraction breakthrough and corresponding temperature at the exit of the PBA for each heat-transfer coefficient. First, it is notable that breakthrough began at an earlier stage for the adiabatic case and occurred later as the heat-transfer coefficient was increased. For higher temperature, the decrease in adsorption equilibrium capacity led the breakthrough to occur quicker as it is shown in Figure 12a. From the temperature curves in Figure 12b, it is visible that the peak became narrower and decayed faster when moving from *h*_w_ = 4.5 to 9.5 W/m^2^ K, promoted by a higher cooling rate by the surrounding. For the adiabatic case, the temperature peak was much larger and was retained for a longer period, as illustrated by Figure 13a–c that shows axial distribution of temperature for each case inside the column, as the breakthrough front reached the column midway. It is apparent in the adiabatic case that since the heat could not be dissipated through the wall, it was dispersed through the flow and associated with a desorption of CO_2_ at reduced equilibrium capacity at peak regions of elevated temperatures.

The impact of the heat-transfer coefficient of the wall in Figure 13 enabled discrimination of the thermal dissipation over the wall from that by gas flow convection in the PBA. High values of heat-transfer coefficient of the wall in Figure 11 reduced the hot zone area at the center of the bed principally by radial cooling, less resistance to heat transfer at the vicinity of the wall, and improved mixing over the larger porosity zones in the vicinity of the wall. The reduced hot zones started from particles with no contact with the wall and cooled off progressively toward the wall. The spatial distribution of energy released from the exothermic adsorption of CO_2_ was then the result of improved radial distribution of the heat but also of the more complex interplay between the fluid flow as contributor to the heat-transfer coefficient of the wall and heat and mass transport rates between zeolite particles and inside zeolite particles. As the adsorption progressed along the bed, the later cooled down, particularly at high values of heat-transfer coefficient.

### 3.3 Model Validation

**Figure 14:**
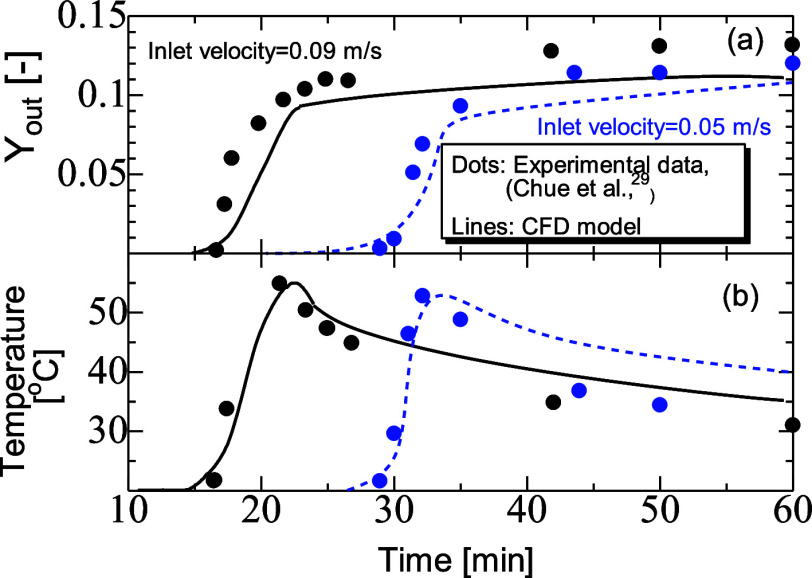
Validation of the breakthrough mole fraction (*Y*_out_) (a) and temperature (b) profiles of CO_2_ on zeolite 13X. PBA of 25 mm ID, 100 mm length, diffusivity of 10^–8^ m^2^/s, AR of 10, inlet temperature of 20 °C, and *h*_w_ of 9.5 W/m^2^ K.

The data of isotherms, breakthrough, and operating conditions (i.e., including bed length, diameter, porosity, inlet gas velocity, vessel pressure, and composition), as illustrated in Table 3, were obtained from the experimental work by Chue et al.,^29^ including an AR of 10 (i.e., 10,490 solid particles) and similar volumetric space velocity value of 0.55 and 0.86 h^–1^ (i.e., inlet velocity of 0.09 and 0.05 m/s, respectively). Figure 14a,b shows mole fraction breakthrough and temperature for both the experimental study by Chue et al.^29^ and for the 3D model. The results show similar trends and can be observed to imitate the experimental study, offering access to the key trends in a transient adsorption in local space. The experimental results showed some differences, most notably, the peak temperature and the height of the breakthrough plateau. Reliable data on the physical properties of the PBA (Table 1) along with a more realistic structure that replicate the adsorption of CO_2_ would have improved the trends of the dispersive flow (i.e., tails of the curves) observed in Figure 14a,b.

**Table 3:** Operation Characteristics of the PBA

	this work	Xue^27^
inlet velocity (m/s)	0.05–0.095	0.132–0.200
bed diameter (m)	0.025	0.0254
bed porosity (−)	0.5	0.32
length (m)	0.3	1
zeolite loading (kg)	0.221	0.38
effective diffusivity (m^2^/s)	10^–8^	10^–8^
surrounding and inlet temperature (K)	293	295
particle size (m)	2.5 × 10^–^^3^	2.5 × 10^–^^3^
bed density (kg/m^3^)	750	750
heat capacity of zeolite (J/kg·K)	920	920
operated pressure (kPa)	110	109.4–122.6
inlet CO_2_ mole fraction (−)	0.15	0.1486
wall heat-transfer coefficient (W/K·m^2^)	9.5	7.5–9.5

**Figure 15:**
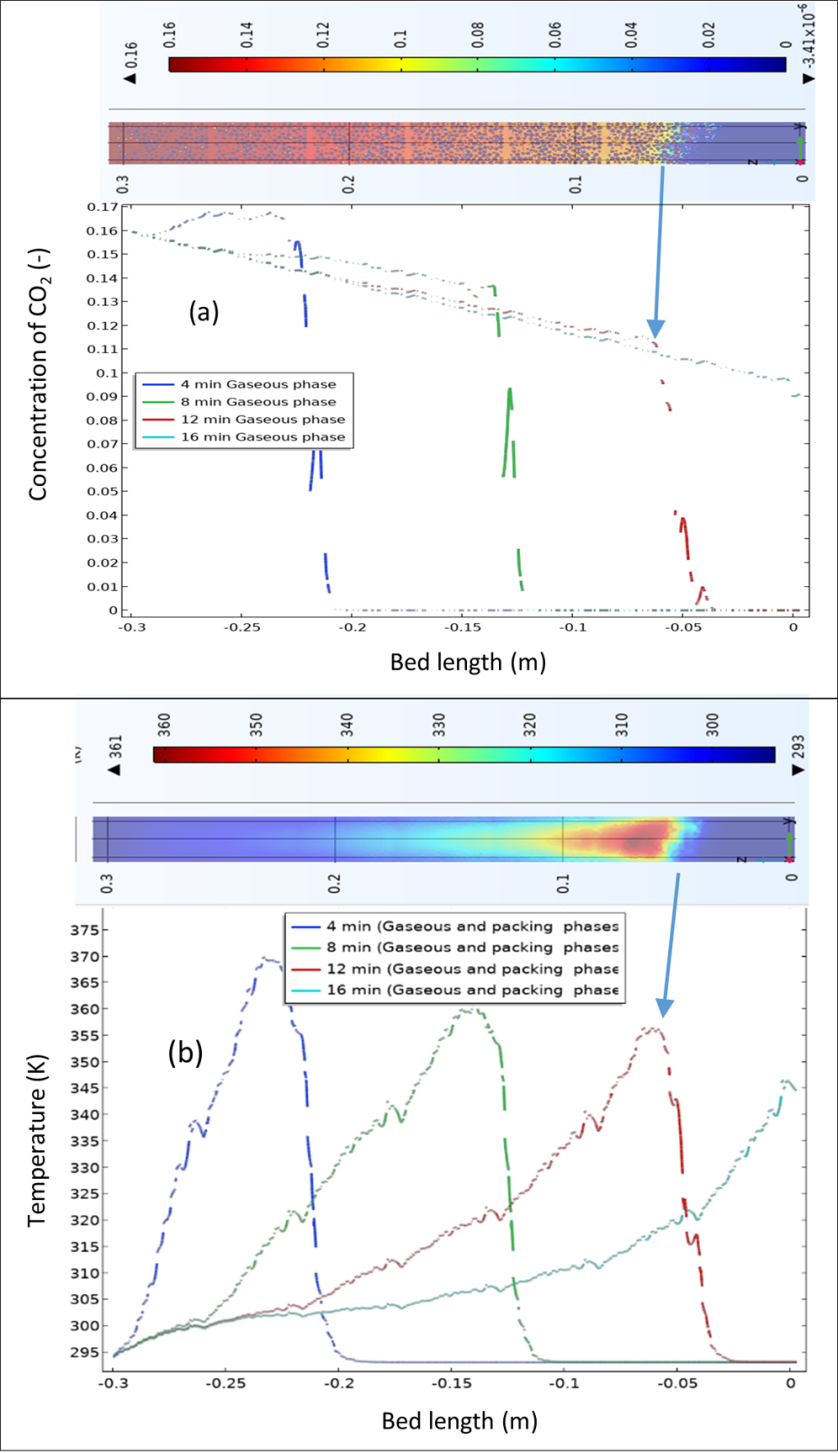
Transient profiles of axial mole fraction of CO_2_, *Y* [—], (a) and temperature (b) along the PBA. Feed inlet velocity of 0.09 m/s and *D*_*i*,s CO2_ of 10^–8^ m^2^/s, 100 mm length, AR of 10, inlet mole fraction of CO_2_ of 0.15, inlet temperature of 20 °C, and *h*_w_ of 9.5 W/m^2^ K.

The profiles of mole fractions and temperature along the bed length, as shown in Figure 15a,b, respectively, confirm the key role of heat- and mass-transfer behaviors during the adsorption process. Unlike differences in temperature between the gaseous phase and the pore of zeolites, those of mole fraction of CO_2_ between the gaseous phase and the pore of zeolites, as well as inside the pores, are significant, highlighting the relevance of both convective and diffusive mass transfers for this case study.

## 4 Conclusions

The use of tube bundle-type adsorbers is highly prominent in current industries of air filters, pollutant control, thermochemical energy storage systems, and atmospheric revitalization of confined spaces. The tube bundle type of adsorbers as technology for large-scale adsorbers such as CO_2_ separations is a promising technology, being facilitated by the linear scaleup via numbering up of the tubes of low AR. The modeling of such thin tubes remains not fully certain because of the experimental constraints on access to local changes in flow, temperature, and concentration. In this work, the focus has been to approach the modeling of CO_2_ adsorption in discrete porous media using the 3D structure and DEM to reproduce as close as possible the realistic operations. Typical PBAs are designed with a reduced mean to reach local adsorption, leading to unpredicted effectiveness distribution along the bed length and oversized approximations to deal with potential uncertainties.

It has been herein possible to access the inside of the PBA during a transient operation mode and helped understand the local breakthrough phenomena without the need of dispersion models, which are generally reliable for dedicated laboratory conditions and uniform mixing characteristics. The simulation was set up to minimize use of the empirical parameters and operate a model that represents as close as possible realistic cases. This was done by adopting an adsorption model that relied only on the equilibrium data (isotherms) and driving forces that relied on mass and heat transfer in the solid and gaseous phases and the boundaries of both. The point at which approximations were made was with the lumping of intraparticle parameters, which can be approached further by a distinct multiscale approach. An approach that includes intraparticle representation of the pore network as close as possible to the realistic textural geometry reduces the use of the averaging approach by the pore network models.

The CO_2_ diffusivity inside the porous zeolite has been investigated over the variation in adsorbent pore size, which in turn affects the transport within the solids. The study confirmed any increase in diffusivity would result in a slow onset of breakthrough, widened MTZ, and prolonged adsorption taking place. Many of the recently developed materials for enhanced adsorption have been focusing on increasing access to the internal pores and tuning pore geometry for increased mobility and selectivity. The results validated literature knowledge on impact of the diffusion in solids by inhibiting the onset of breakthrough, widening the MTZ, and reducing the breakthrough temperature.

The results of the work showed agreement with the experimental results and contributed to access to insights into the behaviors that occur inside the PBA, particularly for long breakthrough time, which was deemed satisfactory as behavior and the trends were the primary focus. Despite this, there is a great deal of room for further investigation into modeling adsorption in packed beds designed as close as possible to the industrially relevant scales by extension to a tube bundle, actual scaling up of the PBA, reduced approximations of heat and mass transfer at gas–solid boundaries, including both the wall as well as the packing, reduced averaging of the solid zeolite structural properties, and dynamic operations under pressure or temperature swinging cycles.

## Supporting Information Available

Discretization assessment (PDF)
Axial sections of concentration of CO_2_ adsorption on zeolite 13X along the PBA (MP4)
Axial sections of temperature for CO_2_ adsorption on zeolite 13X along the PBA (MP4)

## Abbreviations

*a*: ratio of pore surface area to pore volume [m^2^ m^–3^]
*B*: Langmuir isotherm parameter [1/Pa]
*C*_*p*_: heat capacity [J/kg·K]
*c*_i_: concentration of species *i* [mol m^–3^]
*d*_p_: particle diameter [m]
*D*: tube wall diameter [m]
*D*_*i*_: diffusivity [m^2^ s^–1^]
*D*_*i*K_: Knudsen-type diffusion coefficient [m^2^ s^–1^]
*D*_*i*b_: bulk solid-type diffusion coefficient [m^2^ s^–1^]
*h*: heat-transfer coefficient [W m^–2^ K^–1^]
*I*: identity matrix [—]
*k*: thermal conductivity [W m^–1^ K^–1^]
*M*: molecular weight [kg mol^–1^]
*p*_*i*_: partial pressure of species *i* [Pa]
*q*: adsorbed surface quantity [mol kg^–1^]
*q*_s_: surface saturation quantity [mol kg^–1^]
*R*: universal gas constant [J mol^–1^K^–1^]
*R*_adsi_: rate of adsorption [mol m^–2^ s^–1^]
*r*_p_: average radius pore [m]
*S*: surface area per unit mass [m^2^ kg^–1^]
*t*: time [s]
*T*: temperature (K)
*u*: velocity vector [m s^–1^]
*Y*: mole fraction [—]

## Greek Letters

ε_s_: zeolite porosity [—]
Δ*H*_ads_: enthalpy of adsorption [J mol^–1^]
μ: dynamic viscosity [Pa s]
ρ: density [kg m^–3^]
τ_s_: tortuosity [—]

## Subscripts:

ads: adsorption
s: solid phase
g: gaseous phase
Surf: surface
w: wall
