# Supplementary material for: Modeling CO2 Adsorption in a Thin Discrete Packing
Source: Ind Eng Chem Res. 2024 Apr 10;63(16):7321–34. doi: 10.1021/acs.iecr.3c04231 (PMC11046433; doi:10.1021/acs.iecr.3c04231)
Supplement: Supplementary file 1 — ie3c04231_si_001.pdf [file ie3c04231_si_001.pdf]

Modelling CO_2 adsorption in a thin discrete packing

Authors : Michael Wray, Farida Amrouche and Farid Aiouache*
Lancaster University, School of engineering, LA1 4YR, UK

*Corresponding author: e-mail: f.aiouache@lancaster.a.cuk

Discretisation assessment

Potential errors that could be associated with the CFD simulation were examined by following the approach given by Celik et al.,[32] ensuring that the results were free of mesh size. The representative grid size h_0 procedure was defined as:

$$h_0 = \left(\frac{1}{N}\sum_{i=1}^{N} \Delta V_i\right)^{\frac{1}{3}} \quad \text{(A.1.1)}$$

where ΔV is the cell volume and N is the number of cells. Three grid refinement factors higher than 1.3 were used for the ultimate refinements. The apparent order *m* of the method was defined by Equations (A.1.2) to (A.1.4.) :

$$m = \frac{\left| ln \left|\frac{\Theta_{32}}{\Theta_{21}}\right| + q(m) \right|}{ln\,(r_{21})} \quad \text{(A.1.2)}$$

$$q(m) = ln\left(\frac{r_{21}^m - s}{r_{32}^m - s}\right) \quad \text{(A.1.3)}$$

$$s = 1.sgn\left(\frac{\Theta_{32}}{\Theta_{21}}\right) \quad \text{(A.1.4)}$$

where $h_{0,1}<h_{0,2}<h_{0,3}$, $r_{21}=h_{0,2}/h_{0,1}$, $r_{32}=h_{0,3}/h_{0,2}$, $\Theta_{32}=u_3-u_2$, $\Theta_{21}=u_2-u_1$. u_k(k=1, 2, 3) expresses velocity values taken at three grid locations in the packed bed as shown in **Figure A.3** and sgn is the function signum. Equation (A.3.3) was solved numerically for *m*. This was used to find the relative errors (Equations A.3.5 and A.3.6) and the fine grid convergence GCI (Equations A.1.7 and A.1.8)

$$e_a^{21} = \frac{u_1 - u_2}{u_1} \quad \text{(A.1.5)}$$

$$e_a^{32} = \frac{u_2 - u_3}{u_2} \quad \text{(A.1.6)}$$

$$GCI^{21} = \frac{1.25 e_a^{21}}{r_{21}^m - 1} \quad \text{(A.1.7)}$$

$$GCI^{32} = \frac{1.25 e_a^{32}}{r_{32}^m - 1} \quad \text{(A.1.8)}$$

Mov 1 .mp4

Mov 1. Axial sections of concentration of CO_2 adsorption on zeolite 13X along the PBA at t=25 min for (a) h_w=9.5W/m^{2}K, (b) h_w=4.5W/m^{2}K and (c) h_w=0W/m^{2}K (right). PBA of 25 mm ID, 300 mm length, $D_{i,s}$=1x10^{-7} m^2/s, AR of 5, feed inlet velocity of 0.096 m, inlet mole fraction of CO_2 of 0.15 and inlet temperature of 20^{0}C

Mov 2.mp4

Mov 2. Axial sections of temperature for CO_2 adsorption on zeolite 13X along the PBA at t=25 min for (a) h_w=9.5W/m^{2}K, (b) h_w=4.5W/m^{2}K and (c) h_w=0W/m^{2}K (right). PBA of 25 mm ID, 300 mm length, $D_{i,s}$=1x10^{-7} m^2/s, AR of 5, feed inlet velocity of 0.096 m, inlet mole fraction of CO_2 of 0.15 and inlet temperature of 20^{0}C
